# Exploring the spatiotemporal effects of domestic violence in Puerto Rico during the COVID-19 period: a geographically and temporally weighted regression approach

**DOI:** 10.3389/fpubh.2026.1776190

**Published:** 2026-04-28

**Authors:** Phyllis N. Muniu, Jeremis N. Morales Morales, Joshua Atsu, Folashade B. Agusto, Carmen Caiseda, Jarron M. Saint Onge, Atinuke Adebanji

**Affiliations:** 1 Ecology and Evolutionary Biology, University of Kansas, Lawrence, KS, United States; 2 Applied Mathematics, Inter American University of Puerto Rico, San German, Puerto Rico; 3 Mathematics, Kwame Nkrumah University of Science and Technology, Kumasi, Ghana; 4 Mathematics and Applied Mathematics, North-West University, Potchefstroom, South Africa; 5 Sociology and Population Health, University of Kansas Medical Center, Kansas City, KS, United States; 6 Statistics, Purdue University, West Lafayette, IN, United States

**Keywords:** domestic violence, geographically and temporally weighted regression, Moran's I, socioeconomic factors, spatiotemporal heterogeneity

## Abstract

Domestic violence is an epidemic with profound impacts on both the mental and physical well-being of victims. In Puerto Rico, reported cases have risen rapidly in recent years, particularly after the 2020 COVID-19 pandemic. Despite its urgency, domestic violence remains understudied in the context of spatial information science, with limited research exploring how its relationship with sociodemographic factors varies across space and time. This study addresses this gap by investigating the spatiotemporal effects of domestic violence in Puerto Rico from 2020 to 2022, the COVID-19 pandemic period using geographically and temporally weighted regression (GTWR). Police-reported domestic violence data from the thirteen police regions of Puerto Rico showed a decline in 2020. Following the COVID-19 lockdown measures and mobility restrictions, it remains unknown whether this reduction is due to disruptions in reporting access and service availability or a decrease in underlying domestic violence. Results revealed statistically significant spatial clustering of police-reported domestic violence at the police-region level, with San Juan and Bayamón emerging as persistent hotspots. The female population showed strong positive associations (GTWR coefficients ranging from 0.5 to >1.0 across Puerto Rico), while separated populations exhibited moderate to strong positive relationships (0.25 to >1.0), with localized negative associations observed in the regions Aguadilla and Mayaguez. However, the population with moderate income stress demonstrated strong negative associations (coefficients < -1.0). These spatially varying relationships were not captured by the global ordinary least squares model(OLS). Overall, GTWR improved model fit (Δ Adj. *R*^2^ = 0.0907). These results show that socioeconomic associations with domestic violence are spatially and temporally non-stationary, and can help public health officials prioritize region-specific prevention and intervention strategies to guide the effective allocation of public health resources in Puerto Rico.

## 1 Introduction

Domestic violence (DV) is a pervasive social issue that especially affects women across various demographic categories, including race and socioeconomic status (SES). The World Health Organization (WHO) recognizes intimate partner violence (IPV), a specific form of DV, as a global public health concern due to its high prevalence and severe consequences for women's and children's health and well-being. Alarmingly, statistics indicate that approximately one in three women worldwide has experienced some form of violence (1). DV is predominantly perpetrated by men against women, highlighting a gendered aspect of domestic violence (2).

Domestic violence is highly underreported (3) in part due to varied definitions of violence, perpetrator, locations, and based on individual perceptions. The recent COVID-19 pandemic presented additional risks of DV toward women, with government lockdown increasing feelings of entrapment with their abusers, and a potential reluctance to seek help or report incidents, particularly among women with high levels of financial dependency (4).

The COVID-19 pandemic reached Puerto Rico in early 2020, following its global declaration by the World Health Organization in March 2020 (5). In response to the rapid spread of the virus, the Government of Puerto Rico implemented strict island-wide mitigation measures beginning in mid-March 2020 (6). These measures included mandatory curfews, suspension of non-essential business activity, closure of schools and public venues, restrictions on public transportation, and limits on inter-municipal movement (6, 7). As a result, daily routines were substantially altered, with prolonged household confinement and reduced access to public and institutional spaces. While these restrictions were designed to reduce viral transmission and protect public health (8), they also disrupted social interaction patterns, access to services, and formal reporting mechanisms.

The lockdown and social isolation measures, while necessary for public health, have also been linked to increased domestic violence, as individuals were forced into close quarters, heightening existing tensions (9). Accordingly, the COVID-19 pandemic has been described as a “double pandemic” (10) with increased family violence reported globally, exacerbated by economic insecurity, social isolation, and reduced access to support services. Reports indicate a steady increase in divorce rates, with a notable spike following the COVID-19 lockdown likely due to prolonged family interactions (11).

A study conducted by Hongwei (9) in China discussed the economic impact of the pandemic, noting that financial distress and unemployment worsened violence and highlighting the need for economic support systems to mitigate the effects of crises on vulnerable populations. The study also emphasized the role of patriarchal values in Chinese society, which contributed to the normalization of family violence and hindered victims from seeking help. This cultural backdrop complicates the response to domestic abuse during crises. Ohajunwa et al. (4) also explored the interplay between lockdowns, COVID-19 transmission, and domestic violence. The study emphasized the importance of understanding how different lockdown strategies impacted both the spread of COVID-19 and the incidence of domestic violence. The findings suggest that while multiple shorter lockdowns may be safer for domestic violence victims, they do not necessarily control the spread of COVID-19 as effectively as longer lockdowns.

In Bangladesh, Hossain et al. (12) studied the increase in DV against women and children during the COVID-19 pandemic. The study investigated family violence among 511 families. The analysis identified two critical features significantly associated with DV: family income level and education levels during the pandemic. These factors were found to be highly relevant in predicting the likelihood of DV incidents. Some other studies have also analyzed DV trends across different regions and demographics. For example, Evans et al. (13) examined police statistics in Atlanta, Georgia, to understand fluctuations in domestic abuse cases, and the results of the study showed an increase in DV reports during the pandemic compared to the same weeks in previous years. Notably, there was a spike in incidents following the implementation of shelter-in-place orders, suggesting that movement restrictions may have increased DV situations.

In Taiwan, DV cases have doubled in the past decade. This surge has raised serious concerns, particularly given the limited number of social workers available to effectively address these issues. Hsieh et al. (14) emphasize that DV not only affects immediate victims, but also has far-reaching effects on their families, potentially leading to a cycle of violence across generations. The study also introduced the Data for Social Good Initiative, which collaborates with the Taipei City Government to enhance DV prevention strategies. Maposa et al. (15) identified specific districts in Rwanda as hotspots for IPV, particularly in the Northwestern regions. The spatial effects accounted for a significant portion (64%) of the variance in IPV prevalence, notably higher among women (46%) compared to men (18%). While spatial heterogeneity is well documented, large-scale crises further reveal that DV risk also evolves temporally, often intensifying during periods of social and economic disruption (16–18). Natural disasters such as hurricanes and public health emergencies like the COVID-19 pandemic represent distinct but comparable events that alter household dynamics, economic security, and access to protective services (9).

In Puerto Rico, major hurricanes, most notably Hurricane Maria in 2017, resulted in prolonged infrastructure collapse, displacement, and economic hardship, conditions consistently associated with increased IPV in post-disaster settings (19, 20). Studies of hurricane-affected populations show that DV frequently rises in the months following disasters, with effects varying across urban and economically vulnerable regions (21, 22). These hurricane-related stressors preceded the COVID-19 pandemic and likely shaped baseline regional patterns of DV, which were subsequently compounded by pandemic-related lockdowns, social isolation, and financial strain (10, 23).

Yet, despite growing recognition of spatial clustering and crisis-driven surges in domestic violence, the links between region and contextual factors remain limited to specific locales. We extend this research by employing a set of innovative data sets in the Puerto Rican context. This study aims to further examine how sociodemographic and socioeconomic factors are associated with DV across police regions in Puerto Rico, and how these relationships changed over time, using geographically and temporally weighted regression.

The primary objectives of this study are :

(i) Examine geographical and temporal patterns of domestic violence in Puerto Rico, with particular attention to variations across police regions.
(ii) Identify region-specific factors contributing to higher rates of DV, to inform more effective, targeted interventions and prevention strategies.
(iii) Investigate the spatiotemporal relationship between socioeconomic characteristics and domestic violence, identifying regional disparities and underlying determinants using geographical and temporal weighted regression (GTWR).

The remainder of the paper is structured as follows: Section 2 outlines the data and methodology used in the analyses; Section 3 presents the key findings; and Section 4 provides the discussion and conclusions.

## 2 Methodology

### 2.1 Study area

**Figure 1:**
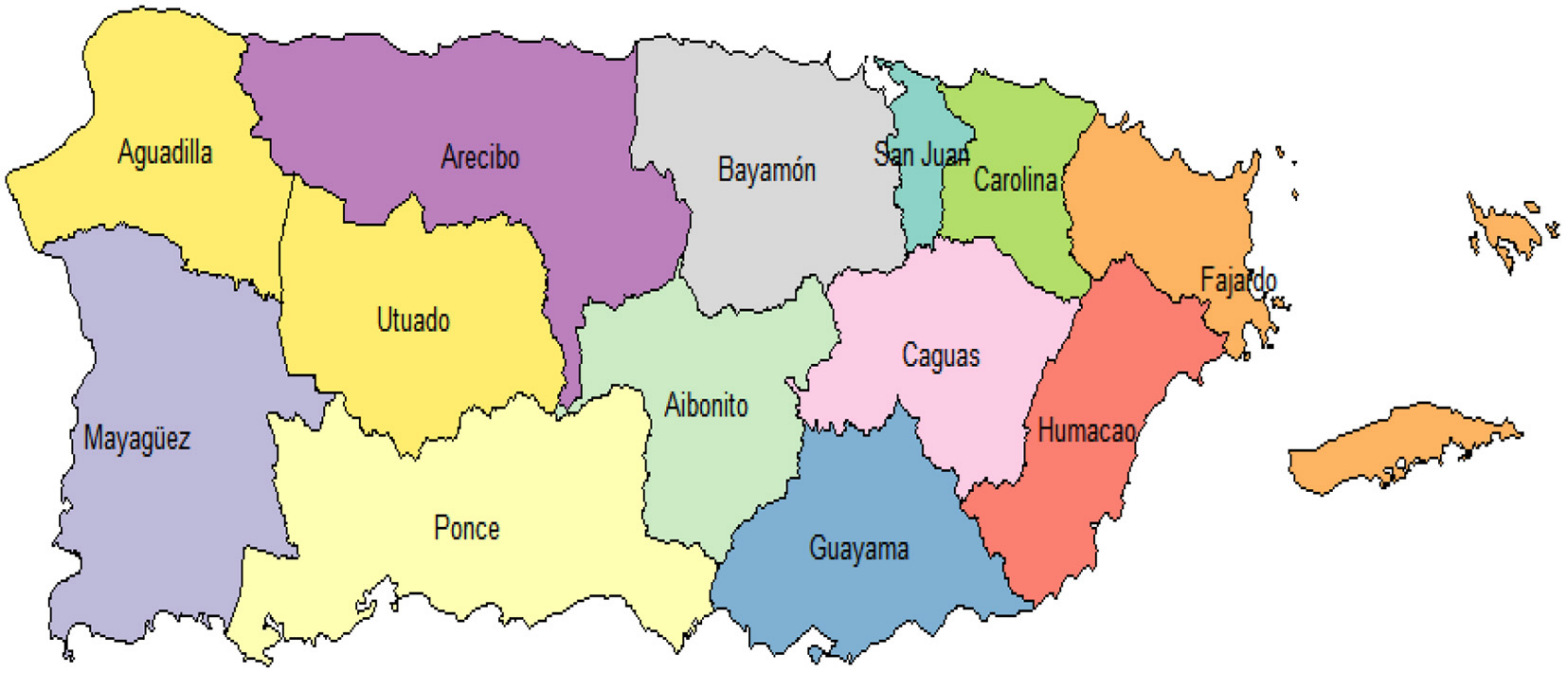
The map of Puerto Rico showing the 13 police regions.

Puerto Rico (PR) located in the northeastern Caribbean at approximately 18.2208° N latitude and 66.5901° W longitude, is an unincorporated territory of the United States. It covers a total land area of 8,870 square kilometers (3,424 square miles) (24). The island is bordered by the Atlantic ocean to the north and the Caribbean sea to the south. Puerto Rico is divided into 78 municipalities, which are further grouped into 13 police regions, as shown in Figure 1, for law enforcement and crime monitoring purposes (25). These police regions, controlled by the PR Police Bureau, are administratively critical in addressing criminal activity, including domestic violence.

### 2.2 Datasets

#### 2.2.1 Domestic violence data

**Figure 2:**
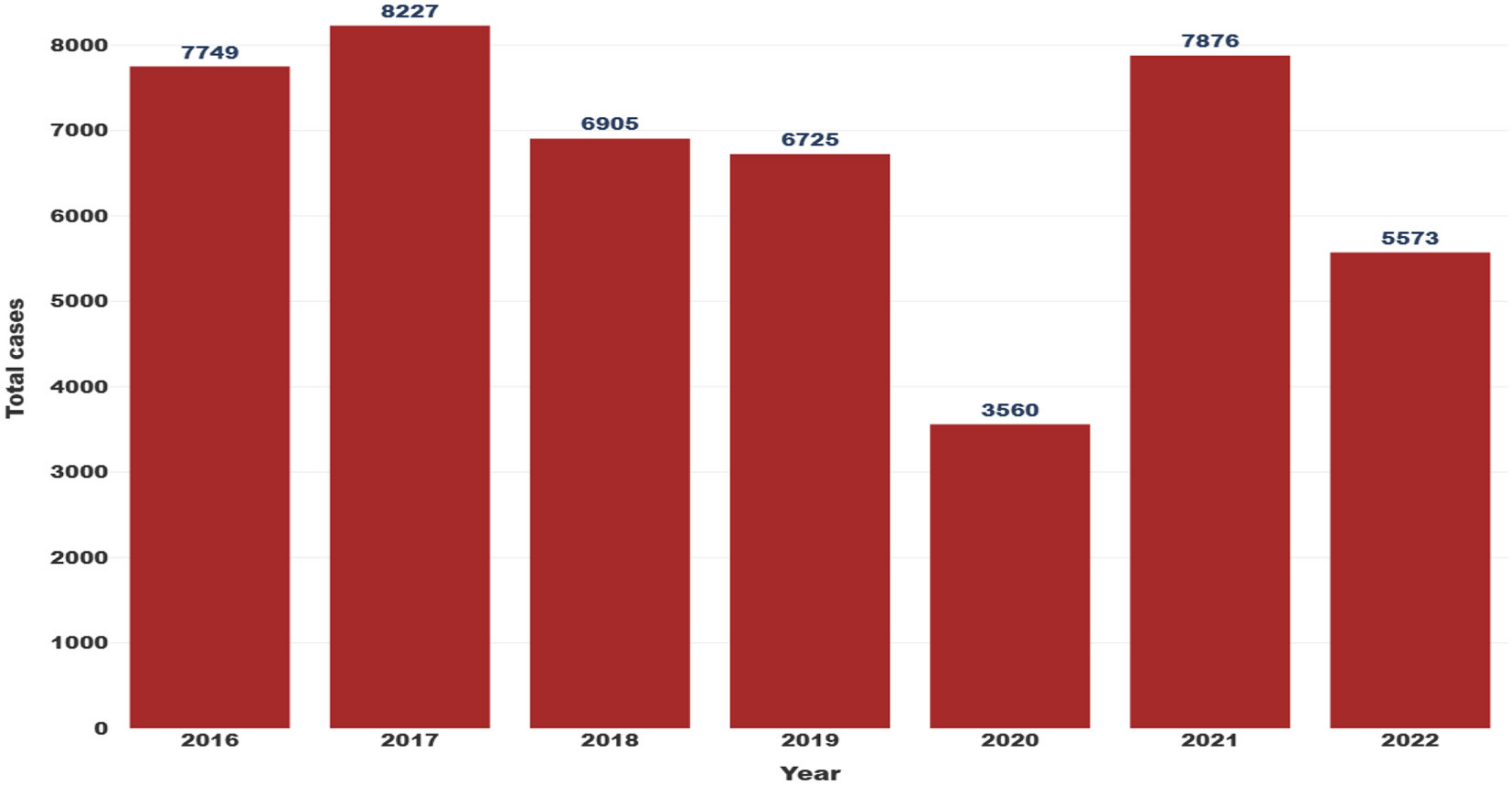
Yearly distribution of the reported domestic violence cases in Puerto Rico's police regions from 2016 to 2022.

To contextualize pandemic-era patterns, Figure 2 presents annual domestic violence case counts from 2016 to 2022, providing a pre-pandemic baseline. Reported cases totaled 7,749 in 2016 and 8,227 in 2017, coinciding with the year of Hurricane Maria, which caused widespread displacement, infrastructure failure, and prolonged socioeconomic disruption across the island. Following this peak, reported cases declined in 2018 (6,905 cases) and 2019 (6,725 cases), suggesting partial stabilization in the post-hurricane period. A pronounced drop in reported domestic violence cases was observed in 2020 (3,560 cases). This decline is likely attributable to underreporting associated with mobility restrictions, social isolation, limited access to reporting mechanisms, and reduced availability of support services, rather than a true reduction in incidence (26). In contrast, reported cases increased sharply in 2021 (7,876 cases), potentially reflecting the relaxation of lockdown measures, delayed reporting, and the release or transfer of backlogged case records (13). Reported cases declined again in 2022 (5,573 cases), suggesting a partial return toward pre-pandemic reporting levels.

For our study, we focused on the COVID-19 period 2020 to 2022 where the total reported cases of domestic violence across police regions in Puerto Rico from January 1, 2020, to December 31, 2022 were 17,009.

The study data from the United States Census Bureau included a shapefile of the municipalities of PR, and the socioeconomic and demographic data sets (27, 28). The analysis focused on the following variables shown in Table 1: the dependent variable, yearly domestic violence cases recorded for each police region, and the independent variables: poverty status, marital status, age distribution, education level, and female population. The data set covered the years 2020 to 2022.

**Table 1:** Description of variables and data sources (61).

Parameter	Description	Source
Poverty status	Population with *low to moderate income stress* having *125*% to *200*% *poverty levels*.	Census Bureau Tables (56)
Age distribution	Categorized as: Population under 20 years, 20–40 years, 40–65 years, and 65+ years.	Census Bureau Tables (57)
Sex(% female)	The proportion of females in the total population.	Census Population Estimates (58)
Education level	The population with no high school diploma and bachelor's or higher education.	Census Population Estimates (59)
Marital status	The population of people in PR police regions who are divorced or separated.	Census Population Estimates (58)
Social isolation	Composite proxy of population-level mobility contraction and household confinement during COVID-19.	Mobility and energy datasets (29–31)
Domestic violence cases	Reported domestic violence cases for each police region.	Government of PR (60, 62)

**Figure 3:**
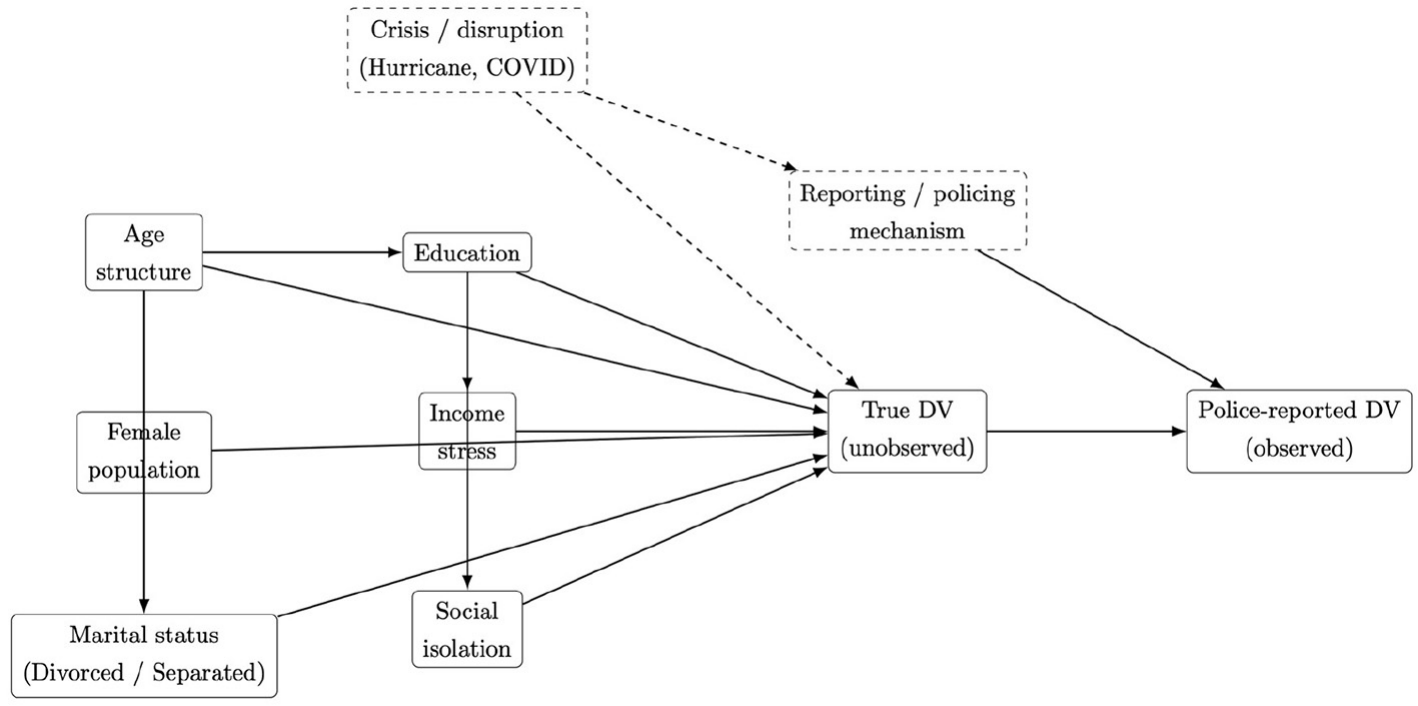
Conceptual directed acyclic graph (DAG) illustrating assumed relationships between socio-demographic factors and *police-reported* domestic violence. The diagram is an interpretive framework (not a causal identification strategy), highlighting measured covariates in the analysis and an unobserved reporting mechanism that may influence observed police-reported counts, particularly during crisis periods.

Figure 3 presents a directed acyclic graph (DAG) that provides a conceptual framework for selecting and interpreting covariates included in our analysis. The DAG was not intended to establish causal effects; rather, clarify assumed structural relationships among measured sociodemographic characteristics, unobserved processes, and the observed outcome, and highlights potential sources of confounding and reporting bias inherent in police-recorded domestic violence data. In the DAG, age structure (20–40 years and 40–65 years) is treated as a foundational demographic characteristic influencing educational attainment, marital status composition, and baseline exposure to domestic violence. The female population represents demographic exposure at the regional level, reflecting the disproportionate burden of domestic violence experienced by women. Education, operationalized as the proportion without a high school diploma and the proportion with a bachelor's degree or higher, is conceptualized as a central socioeconomic characteristic that influences both income stress and social isolation. Income stress, defined as households with income between 125% and 200% of the federal poverty level, captures moderate economic vulnerability that may be associated with relational strain. Social isolation reflects reduced social connectedness and limited access to support networks, and is conceptualized as influencing both underlying domestic violence risk and reporting behavior. Marital status (divorced and separated populations) represents relationship instability that may be associated with domestic conflict at the population level. The node labeled true domestic violence represents the underlying occurrence of domestic violence incidents, which is unobserved. The observed outcome in this study, police-reported domestic violence, captures only incidents that become formally recorded. The reporting/policing mechanism is modeled as an unobserved process influencing whether true incidents are recorded in police data. Accordingly, the models used adjusted for measured sociodemographic characteristics identified in the DAG as potential confounders of spatial temporal associations. However, unmeasured factors and reporting mechanisms may still influence observed police-reported cases. Estimated coefficients were therefore interpreted as conditional associations rather than causal effects.

#### 2.2.2 Social isolation index

Governments have implemented a variety of measures, including lockdowns (i.e., stay-at-home orders) and social distancing guidelines, to influence human behavior toward predictable consequences as a trade-off to mitigating the spread of the virus. Due to the restrictions on movement, there were differential consequences for electricity consumption, hence causing economic distortions. To characterize the intensity of population-level mobility restriction and household confinement during the COVID-19 period, we constructed a composite index using (i) Google Community Mobility Reports, (ii) Puerto Rico electricity consumption, and (iii) Facebook Data for Good movement-range metrics. We use the term “isolation index” as a contextual proxy for reduced mobility and increased time at home during lockdown periods. Notably, it does not measure interpersonal isolation, loneliness, or household-level social dynamics, and the observational design does not support causal interpretation.

Both Google mobility reports and Facebook's movement range maps consist of aggregated anonymized data from mobile devices and offer a valuable resource to analyze real-time changes and explore changes in mobility patterns during the pandemic. With electricity consumption, we can confirm one of the consequences of shifting mobility patterns during the pandemic, therefore, causing socioeconomic disruptions in the long run.

Google mobility reports provide insights into how people's movement patterns have changed in response to COVID-19 (29). These reports track mobility trends over time across various locations, helping governments, public health officials, and researchers understand the impact of lockdowns, social distancing, and other recommended public health measures. The reports indicate the change in foot traffic compared to a baseline period. Each category shows a percentage change from the baseline, which was the median value for the corresponding day of the week during the 5-week period from January 3 to February 6, 2020. A positive percentage indicates increased mobility compared to the baseline, while a negative percentage show decreased mobility. The following key categories and data contained within the reports are the following:

(i) Retail and recreation: Tracks visits to places like restaurants, cafes, shopping centers, theme parks, museums, libraries, and cinemas.
(ii) Grocery and pharmacy: Monitors foot traffic to grocery stores, markets, farmers markets, specialty food shops, drug stores, and pharmacies.
(iii) Parks: Measures visits to national parks, public beaches, marinas, dog parks, plazas, and public gardens. It captures how much time people spend in outdoor recreational spaces.
(iv) Transit stations: Tracks movement around public transport hubs such as subway, bus, and train stations.
(v) Workplaces: Measures mobility trends in office buildings and workplaces. This highlights the shift toward remote work or office closures.
(vi) Residential: Tracks the time people spend in residential areas. An increase in this metric can indicate that people were staying home more, often due to lockdowns or stay-at-home orders.

The reports are provided for countries, regions, and subregions (for instance, states, provinces, and cities). This allows for localized insights into how mobility has changed in specific areas. Using this data, it is possible to comprehend the effectiveness of social distancing recommendations and gauge economic changes. In this study, mobility reductions in non-residential categories and increases in residential time are interpreted as proxies for mobility restriction and reduced access to public/social spaces.

Puerto Rico's electricity consumption data set typically provides data on the amount of electricity consumed across various time periods within Puerto Rico (30). This data is valuable for analyzing trends in energy usage, forecasting demand, understanding the impact of events like hurricanes, the pandemic, and supporting energy planning. Data is available in monthly time intervals, highlighting consumption patterns. Puerto Rico's main electricity provider is the PR Electric Power Authority (PREPA) and current Transmission, Substation and Distribution infrastructure is administered by LUMA Energy Co.

The PR electricity consumption data set is divided by the following sectoral areas:

(i) Residential: Data on electricity consumption by households.
(ii) Commercial: Covers electricity use in businesses, offices, and other commercial establishments.
(iii) Industrial: Tracks energy usage in manufacturing plants and other industrial facilities.
(iv) Public sector: Includes data on electricity consumption by government buildings, schools, hospitals, and other public infrastructure.
(v) Agricultural: Includes electricity consumed by farmers and ranchers.

The data shows that residential areas increased in consumption while non-residential areas decreased in consumption after lockdown measures were implemented. Feature engineering was deployed with the electricity data. Percentages were calculated for each data point from year 2020 onward using a seasonal baseline curve. A positive percentage represents more consumption than the baseline, while negative values represents the contrary. We interpret increases in residential electricity consumption (relative to seasonal baseline) as a proxy for increased household presence and activity during stay-at-home periods, acknowledging that consumption also reflects weather, appliance use, and other non-mobility factors.

The Facebook “Data for Good” initiative aims to use aggregated, anonymized data to support humanitarian causes and inform public health responses, especially during the COVID-19 pandemic. The movement range maps focus on tracking population movement patterns at various geographic levels, from countries to regions (31). It measures changes in movement by comparing the mobility of users over time to a pre-defined baseline in February 2020.

Two main types of metrics are tracked:

(i) Stay-at-home metrics: This measures how many people stayed within small geographical areas (often interpreted as residential areas), indicating compliance with stay-at-home orders. Defined as single tile users.
(ii) Movement between areas: This tracks movements between regions (for example, movement between different districts or neighborhoods), reflecting how much and how far people were traveling. Defined as multiple tile users.

The movement range data are available at various levels of granularity such as countrywide, statewide and regional including counties and municipalities. Facebook movement-range metrics (single-tile vs. multiple-tile users) proxy the contraction of routine movement at the population level during lockdowns.

### 2.3 Methods used

All basic statistical analyses were conducted in R version 4.1.3, while GeoDa was employed to assess spatial autocorrelation. The model and procedures applied in this study were implemented using an open-source Python library available on GitHub. In particular, we used the geographically and temporally weighted regression (GTWR) repository maintained by Sunkun (32).

#### 2.3.1 Isolation index

An Isolation Index was derived by combining the three aforementioned metrics. To create the Isolation Index, the input values were transformed using a natural logarithm and a binary category was describing if the variables indicate or do not indicate isolation. For Google Mobility (*M*), residential category favored the isolation, while the other categories did not. For electricity (*E*), an increased residential consumption indicated isolation, while commercial consumption did not. Lastly, in Facebook Movement Range (*R*), Stay-at-home favored isolation while movement between areas did not. Considering these, the Isolation Index is represented by Equation 1:


$$\begin{array}{l} I=\overset{3}{\underset{i=1}{\sum }}\beta_{i}^{R}\ln\left(X_{i}^{R}\right)+\overset{3}{\underset{i=1}{\sum }}\beta_{i}^{C}\ln\left(X_{i}^{C}\right) \end{array}$$ (1)

where β_*n*_ ∈ {−1, 1}, $\beta_{n}^{R}$ = Residential parameter = 1, $\beta_{n}^{C}$ = Commercial/non-residential parameter = −1, *M*^*R*^ = residential Mobility, *M*^*C*^ = commercial Mobility, *E*^*R*^ = residential Electricity consumption, *E*^*C*^ = commercial Electricity consumption, *R*^*R*^ = Stay-at-home movement Range, *R*^*C*^ = Commercial or non-residential movement Range, *i* represents the correspondent metric for both residential and non-residential, and X is the raw value calculated from the dataset.

**Figure 4:**
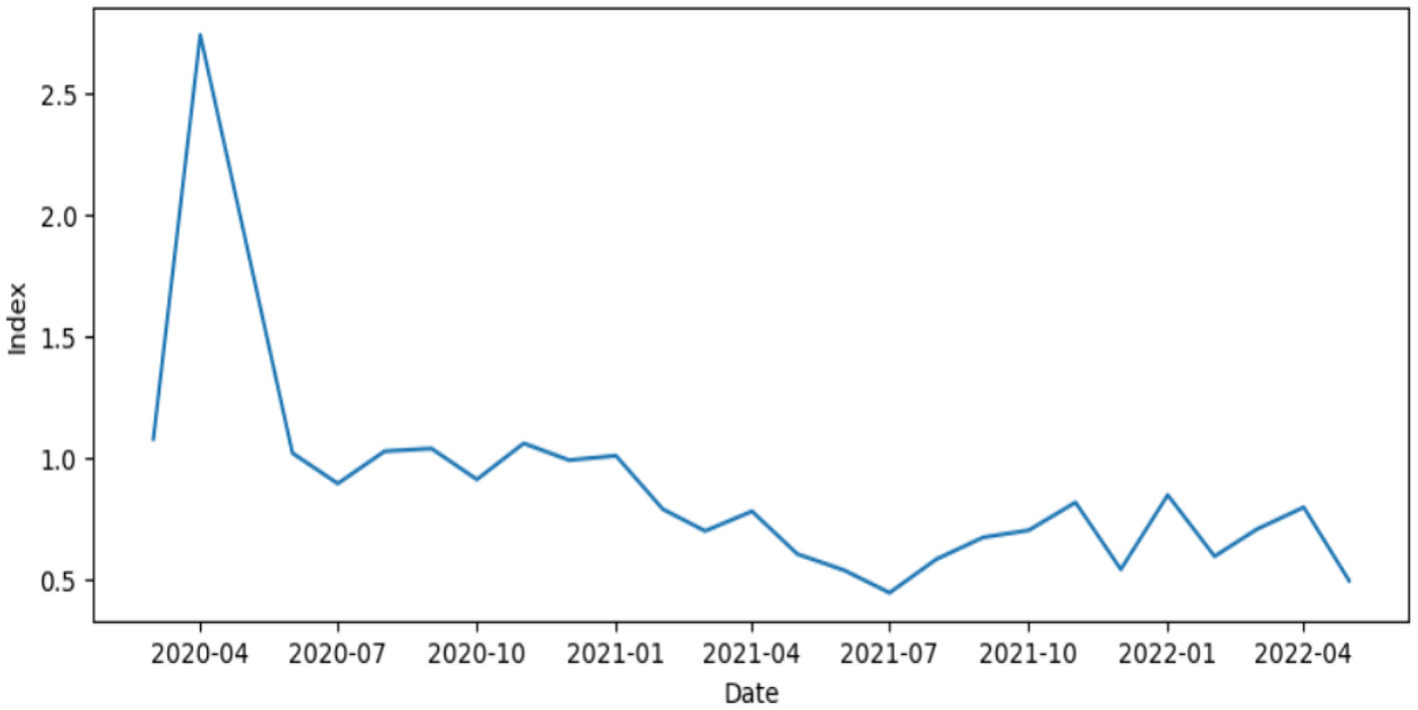
Isolation index for Puerto Rico (PR).

For Puerto Rico data, the isolation index function is shown in Figure 4, showing a maximum that matches the dates of the mitigation strategies. These executive orders initiated with a curfew (Exec. Order No. OE-2020-023, 2020) on March 12, 2020 and escalated into a lockdown (Exec. Order No. OE-2020-029, 2020) by April 10, 2020. Because no individual-level measure of social isolation is available, the index cannot be validated as a direct measure of interpersonal isolation or loneliness. We therefore assessed internal consistency by examining correlations among the three standardized components, and external plausibility by reporting the correlation between *I* and monthly reported DV cases. These checks are descriptive and do not establish causality.

The timing of the peak in our isolation index is consistent with the period of strongest non-pharmaceutical interventions (33). In their reconstruction of Puerto Rico's COVID-19 policy timeline, the government's early response included a declared state of emergency with immediate closures beginning on March 12, 2020, followed shortly by curfews and progressively more restrictive measures as case detection expanded. This aligns with our index reaching its maximum during the initial lockdown phase, when mobility outside the home contracted most sharply and stay-at-home behavior intensified. Importantly, the research (33) notes that by mid-June, when executive orders became more flexible, the epidemiological curve increased an inflection that corresponds to the post-peak decline in our index as mobility restrictions eased.

To assess the empirical relevance of the isolation index, we examined its bivariate association with domestic violence incidence. A negative correlation was observed between the isolation index and domestic violence cases (*r* = −0.53, *p* = 0.0005), supporting its inclusion as a meaningful explanatory variable in subsequent spatiotemporal modeling.

#### 2.3.2 Spatial modeling

To ensure comparability across different variables, all numerical data were normalized (34) between 0 and 1 after getting the rates using Equation 2


$$\begin{array}{l} z=\frac{x-\mu }{\sigma } \end{array}$$ (2)

where *x* is the original data value, μ is the mean and σ is the standard deviation.

#### 2.3.3 Spatial autocorrelation analysis

To examine spatial dependence in domestic violence cases, the local Moran's I test was performed to identify spatial clusters or hotspots of domestic violence incidence. The global Moran's I (35) statistic was used to assess the overall spatial autocorrelation across the study region. Moran's I ranges from −1 (perfect dispersion) to 1 (perfect clustering), with values near zero indicating spatial randomness. It was calculated using Equation 3


$$\begin{array}{l} I=\frac{N\sum w_{ij}\left(x_{i}-\bar{x}\right)\left(x_{j}-\bar{x}\right)}{S_{0}\sum {\left(x_{i}-\bar{x}\right)}^{2}} \end{array}$$ (3)

where *N* is the number of observations, *x*_*i*_ and *x*_*j*_ are the values at locations *i* and *j*, $\bar{x}$ is the mean, *w*_*ij*_ is the spatial weight, and *S*_0_ is the sum of all spatial weights.

The sensitivity of spatial autocorrelation to the choice of spatial weights was evaluated by comparing Queen contiguity, Rook contiguity, and *k*-nearest neighbor (kNN, *k* = 4) specifications in Table 2. Given the similar Moran's I values obtained using Queen and Rook contiguity and the consistently statistically non-significant results under kNN weights, Queen contiguity (order = 1) was used for the analysis as it best captured adjacency-based spatial interaction among irregularly shaped police regions. Local Moran's I was used to identify clusters and spatial outliers in domestic violence incidence. Significance and cluster maps were generated in GeoDa (36) to visualize spatial dependence.

**Table 2:** Global Moran's I statistics for domestic violence rates across police regions in Puerto Rico from 2020 to 2022, using Queen contiguity, Rook contiguity, and K-nearest neighbors (*k* = 4) spatial weight matrices.

Method	Moran's I	*P*-value	Year
Rook	0.1278	0.0915	2020
Rook	0.1717	0.0642	2021
Rook	−0.0484	0.4132	2022
Queen	0.1278	0.0915	2020
Queen	0.1717	0.0642	2021
Queen	−0.0484	0.4132	2022
KNN (*k* = 4)	−0.0133	0.3134	2020
KNN (*k* = 4)	−0.0177	0.3332	2021
KNN (*k* = 4)	−0.1218	0.6051	2022

#### 2.3.4 Ordinary least squares (OLS) regression

Ordinary least squares (37) regression was applied as a baseline model calculated using the following Equation 4


$$\begin{array}{c} y=\beta_{0}+\sum \beta_{k}x_{k}+\varepsilon \end{array}$$ (4)

where *y* is the dependent variable (domestic violence cases), *x*_*k*_ represents the independent variables, β_0_ is the intercept, β_*k*_ are the coefficients, and ε is the error term.

#### 2.3.5 Geographical and temporal weighted regression (GTWR)

To account for both spatial and temporal variations in domestic violence trends, GTWR was applied (38). This method extends geographically weighted regression (GWR) (39) by incorporating a time-dependent weight matrix, allowing relationships to evolve over time. The GTWR model Equation 5 is given by


$$\begin{array}{c} y_{i}\left(t\right)=\beta_{i0}\left(t\right)+\sum \beta_{ik}\left(t\right)x_{ik}+\varepsilon_{i} \end{array}$$ (5)

where *y*_*i*_(*t*) is the dependent variable in location *i* and time *t*, *x*_*ik*_ is the independent variable at location *i* and time *t*, β_*i*0_(*t*) is the intercept, β_*ik*_(*t*) is the local regression coefficient, and ε_*i*_ is the error term. GTWR employs a spatiotemporal weighting function, where the total weight is the product of a spatial weight and a temporal weight. The spatial weight follows a fixed Gaussian kernel function Equation 6


$$\begin{array}{c} w_{ij}^{S}=\exp\left(-\frac{d_{ij}^{2}}{2b^{2}}\right) \end{array}$$ (6)

where *d*_*ij*_ is the distance between the locations *i* and *j*, and *b* is the fixed spatial bandwidth. The temporal weight follows an exponential decay function Equation 7:


$$\begin{array}{c} w_{ij}^{T}=\exp\left(-\frac{\left|t_{i}-t_{j}\right|}{h}\right) \end{array}$$ (7)

where *t*_*i*_ and *t*_*j*_ are the time points for the observations *i* and *j*, and *h* is the temporal bandwidth. The GTWR coefficients were estimated using the following Equation 8


$$\begin{array}{c} \hat{\beta_{i}}\left(t\right)={\left(X^{T}W_{i}\left(t\right)X\right)}^{-1}X^{T}W_{i}\left(t\right)y \end{array}$$ (8)

where *X* is the independent variable matrix, *y* is the dependent variable vector, and *W*_*i*_(*t*) is the spatiotemporal weight matrix. The optimal bandwidth for both space and time was selected by minimizing the corrected Akaike information criterion (AICc) Equation 9, given by:


$$\begin{array}{c} AICc=2n\ln\left(\sigma \right)+n\ln\left(2\pi \right)+n\left(\frac{n+\text{tr}\left(S\right)}{n-2-\text{tr}\left(S\right)}\right) \end{array}$$ (9)

where σ is the standard deviation of the residuals and tr(*S*) is the trace of the hat matrix.

### 2.4 Statistical analysis

**Figure 5:**
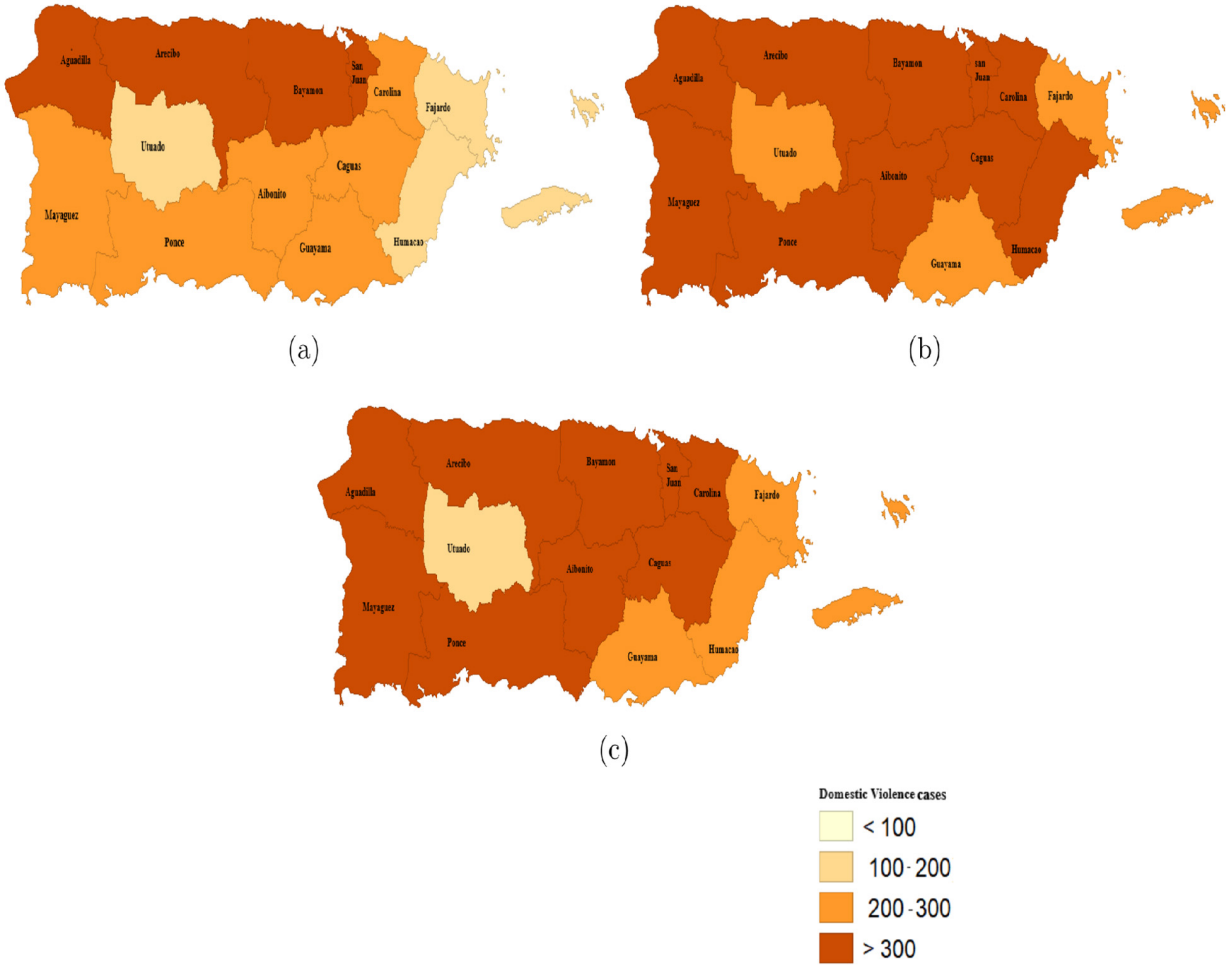
The spatial distribution pattern of domestic violence cases in Puerto Rico's Police Regions 2020–2022. **(a)** reported domestic violence cases for the year 2020; **(b)** reported domestic violence cases for the year 2021; **(c)** reported domestic violence cases for the year 2022.

Figures 5a–c show the spatial distribution of the reported domestic violence cases. The plotted quantile maps for the study period were used to illustrate the concept. The maps revealed an upward trend in domestic violence cases. Notably, the number of police regions reporting fewer than 100 cases decreased, while those exceeding 300 cases had substantially increased. Urban police regions, such as San Juan and Bayamón, consistently had high reported incidence throughout the study, likely due to greater population densities and more accessible reporting mechanisms. However, rural regions like Utuado, Fajardo, and Guayama, recorded fewer than 300 cases each year of the study period. Utuado consistently had the lowest number of reported DV cases indicating either a genuinely lower incidence or potential underreporting due to limited law enforcement presence and fewer support services (40).

**Figure 6:**
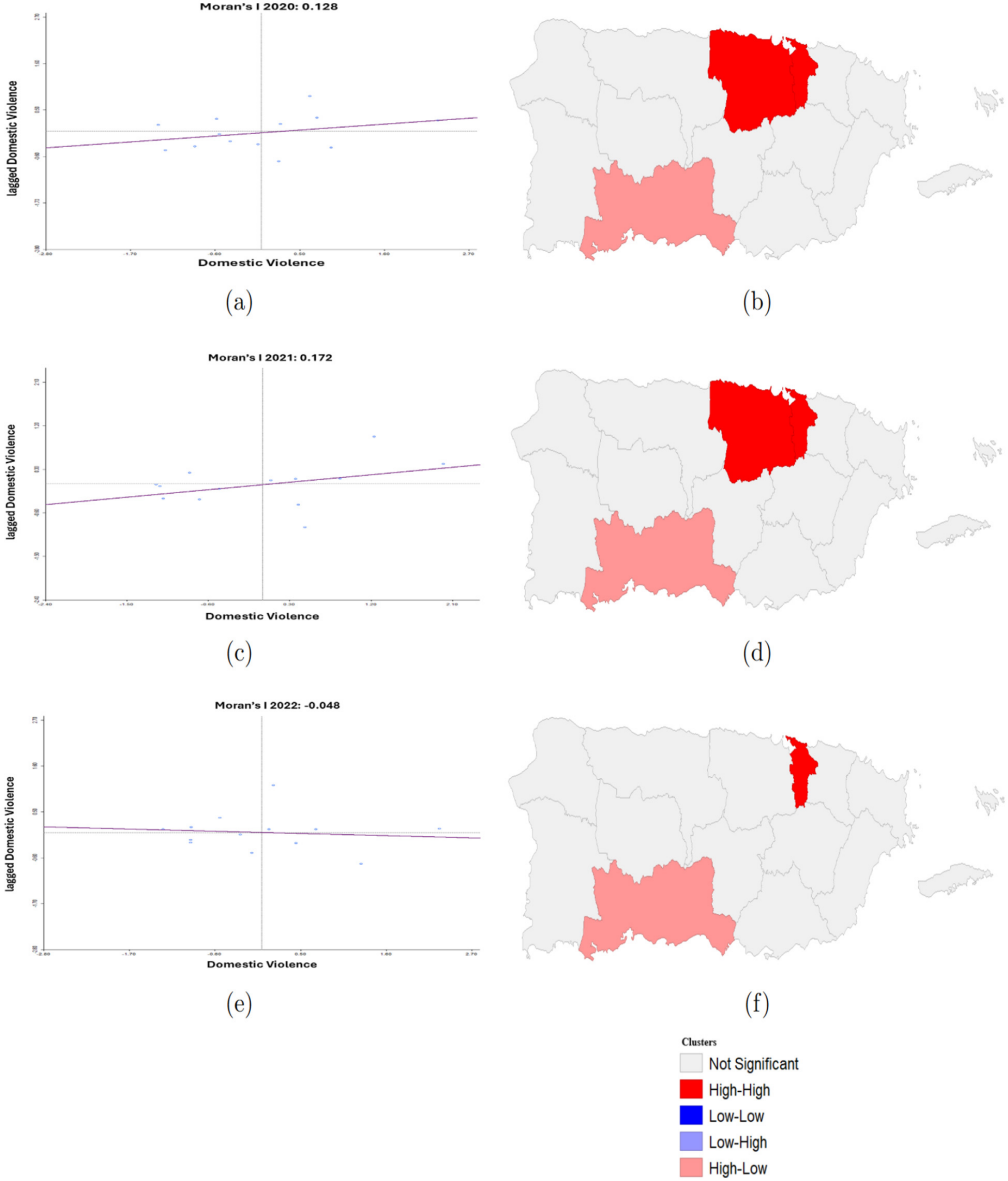
Spatial autocorrelation of domestic violence cases illustrating both global and local spatial patterns across police regions in Puerto Rico from 2020 to 2022. **(a)** the global Moran's I statistic for 2020; **(b)** shows the corresponding Local Moran's I cluster map for 2020, identifying hotspots (High-High), coldspots (Low-Low), and spatial outliers (High-Low, Low-High); **(c, d)** display the global Moran's I and cluster map for 2021, respectively; and **(e, f)** present the global Moran's I and cluster map for 2022.

To evaluate the spatial autocorrelation of domestic violence cases in Puerto Rico from 2020 to 2022, both global and local measures of spatial autocorrelation were employed using Moran's I statistics (41, 42). A global Moran's I test was first conducted to detect the overall spatial autocorrelation in domestic violence cases across the police regions. The Moran's I scatter plots for each year are presented in Figures 6a, c, e. The slope of the regression line in these plots represents Moran's I value, indicating the degree and direction of spatial autocorrelation.

In 2020, the Moran's I was 0.128, indicating a statistically significant positive spatial autocorrelation. This suggests that the police regions with similar DV rates were modestly clustered geographically. In 2021, the value increased to 0.172, revealing a stronger and more structured spatial clustering of DV cases. By contrast, in 2022, the value dropped to −0.048, suggesting a random spatial distribution with no global spatial autocorrelation present.

To explore spatial patterns at the local level, the local Moran's I (LISA) was applied, and the results are shown as cluster maps in Figures 6b, d, f. These maps highlight statistically significant spatial clusters and spatial outliers of domestic violence cases. High-High clusters (red) represent hotspots, where regions with high cases of domestic violence are surrounded by similarly high-cases regions, while Low-Low clusters (blue) represent coldspots of low rates surrounded by low-rate neighbors. High-Low outliers (orange) indicate regions with high case rates adjacent to low-rate neighbors, whereas Low-High outliers (purple) represent regions with low case rates surrounded by high-rate neighbors.

In 2020, the police regions San Juan and Bayamon appeared as a High-High cluster (red), indicating high DV rates surrounded by other high-rate regions. Ponce was identified as a High-Low spatial outlier (pink), meaning it had high DV rates surrounded by police regions with low rates. In 2021, San Juan and Bayamon remained a High-High cluster, and Ponce again appeared as a High-Low outlier, while the rest of the regions showed no statistically significant local spatial autocorrelation. In 2022, a shift in clustering was observed. San Juan was the only hotspot and the Ponce region remained as a High-Low outlier. Most regions were statistically non-significant, reinforcing the global Moran's I finding of spatial randomness in that year.

#### 2.4.1 Sensitivity analysis

Since the outcome of our analysis was based on police-reported domestic violence (DV) cases, we conducted a scenario-based sensitivity analysis to assess robustness to potential underreporting during crisis periods. We considered three underreporting scenarios in which observed police-reported counts were assumed to represent (1 − *x*) of the underlying reported volume, with *x* ∈ {0.10, 0.25, 0.40}. For each scenario, the outcome was adjusted by Equation 10


$$\begin{array}{c} Y^{\left(x\right)}=\frac{Y}{1-x} \end{array}$$ (10)

while all covariates were held unchanged, and the OLS and GTWR models were refit using the same specifications as the baseline analysis. Robustness was assessed by (i) coefficient direction stability (percent of regions retaining the same sign as baseline) and (ii) changes in the mean coefficient magnitude relative to the baseline model.

## 3 Results

This section presents findings sequentially, beginning with sensitivity analyses addressing potential underreporting, followed by predictor selection diagnostics, global OLS estimation, and spatiotemporal GTWR modeling with accompanying model diagnostics and performance comparison.

### 3.1 Sensitivity results

Table 3 shows that coefficient directions were fully stable across all underreporting scenarios: for every predictor, 100% of police regions retained the same coefficient sign as the baseline model. This indicated that the qualitative conclusions regarding the direction of association between covariates and police-reported DV were robust to plausible levels of underreporting. Overall, the scenario analysis supported that the spatially varying associations reported by GTWR were not driven solely by reporting under these plausible underreporting levels.

**Table 3:** Sensitivity of mean coefficients to assumed underreporting in police-reported domestic violence counts.

Variable	Percent same sign	Underreporting	Mean coef (baseline)	Mean coef (scenario)
Age 20–40 years	100%	0.10	1.7421	1.9357
Age 20–40 years	100%	0.25	1.7421	2.3229
Age 20–40 years	100%	0.40	1.7421	2.9036
Age 40–65 years	100%	0.10	−0.093	−0.1034
Age 40–65 years	100%	0.25	−0.093	−0.1240
Age 40–65 years	100%	0.40	−0.093	−0.1550
Bachelor's or higher	100%	0.10	0.2749	0.3055
Bachelor's or higher	100%	0.25	0.2749	0.3666
Bachelor's or higher	100%	0.40	0.2749	0.4582
Female population	100%	0.10	0.9269	1.0299
Female population	100%	0.25	0.9269	1.2358
Female population	100%	0.40	0.9269	1.5448
Divorced population	100%	0.10	0.3913	0.3548
Divorced population	100%	0.25	0.3913	0.4257
Divorced population	100%	0.40	0.3913	0.5321
Separated population	100%	0.10	0.8265	0.9184
Separated population	100%	0.25	0.8265	1.1021
Separated population	100%	0.40	0.8265	1.3776
Isolation index	100%	0.10	0.2468	0.2742
Isolation index	100%	0.25	0.2468	0.3290
Isolation index	100%	0.40	0.2468	0.4113
Moderate income stress	100%	0.10	−4.802	−5.3356
Moderate income stress	100%	0.25	−4.802	−6.4027
Moderate income stress	100%	0.40	−4.802	−8.0034

Counts were adjusted as *Y*^(*x*)^ = *Y*/(1 − *x*) with *x* ∈ {0.10, 0.25, 0.40} and the models refit using the same specification as the baseline. “Percent same sign” indicates the proportion of regions where the coefficient retained the same sign as the baseline model.

### 3.2 Predictor selection

The results from the Variance Inflation Factor (VIF), which test for multicollinearity among the explanatory variables, are presented in Table 4. All VIF values were below the commonly accepted threshold of 10, indicating low multicollinearity and confirming the suitability of the variables for further modeling.

**Table 4:** Variance inflation factor (VIF).

Variable	VIF
20–40 Years	1.544735
40–65 Years	1.709211
No high school diploma	5.809421
Bachelor's degree or higher	4.812565
Female population rate	1.609155
Divorced population	6.570483
Separated population	1.301188
Moderate income stress	6.978550
Social isolation index	6.540339

To assess the stability of predictor selection, a LASSO regression (43) was conducted as a sensitivity analysis using standardized covariates as shown in Table 5. The optimal penalty parameter (α = 0.0077) was selected via cross-validation. The majority of predictors retained non-zero coefficients, indicating stable variable selection and supporting the robustness of the primary specification. The coefficient for the proportion of individuals without a high school diploma was shrunk to zero under penalization, suggesting comparatively weaker independent contribution in the presence of other correlated socioeconomic variables. This led us to continue our analysis without the variable.

**Table 5:** LASSO regression results from sensitivity analysis using standardized predictors.

Predictor	LASSO Coefficient
20–40 years	−0.157
40–65 years	0.190
Bachelor's degree or higher	0.639
No high school diploma	0.000
Female population	0.254
Divorced population	0.265
Separated population	0.277
Isolation index	−4.194
Moderate income stress	0.560
Model diagnostics	
Optimal α	0.0077
RMSE	0.653

The optimal penalty parameter was selected via 10-fold cross-validation. A zero coefficient indicates that the variable was excluded under penalization.

### 3.3 OLS and GTWR results

Table 6 presents the results of the OLS model. The statistically significant predictors of reported domestic violence cases included: the percentage of the population with a bachelor's degree or higher; the female population; The social isolation index; and the population with moderate income stress. The variables exhibited a statistically significant positive relationship with domestic violence cases except the social isolation index which had a statistically significant negative association. The remaining variables, including age groups and marital status categories, were not statistically significant predictors in the model. Heteroscedasticity in the OLS residuals was assessed using the Breusch–Pagan test (44), which yielded a test statistic of 7.2036 with a p-value of 0.5148, indicating no evidence of heteroscedasticity.

**Table 6:** OLS coefficients and significance.

Variable	Coef.	Std. Err.	z	*P*-value
Intercept	1.923	0.660	2.912	0.007^**^
Age 20–40	−0.0816	0.164	−0.497	0.623
Age 40–65	0.2413	0.142	1.696	0.100
Bachelor's degree or higher	0.8689^**^	0.294	2.960	0.006^**^
Female population	0.3146^**^	0.126	2.488	0.019^**^
Divorced population	1.1584	1.176	0.985	0.333
Separated population	0.2659	0.150	1.777	0.086
Moderate income stress	0.8143^**^	0.316	2.578	0.015^**^
Social isolation index	−4.7755^***^	0.899	−5.312	0.000^***^

^*^p < 0.05,

^**^p < 0.01,

^***^p < 0.001.

**Figure 7:**
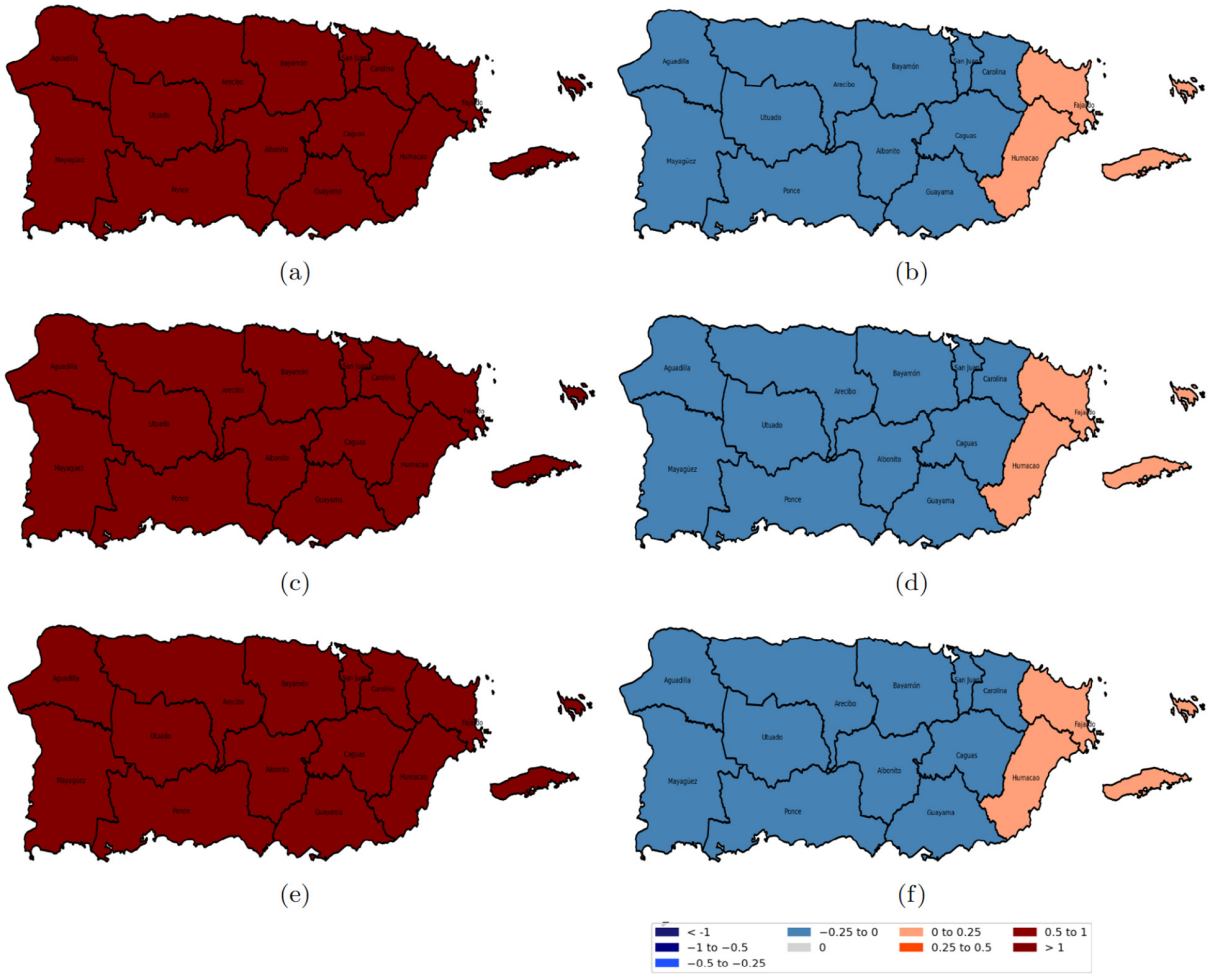
Spatial variation of the estimated coefficients for age groups from 2020 to 2022. Red areas indicate a positive effect of the age group on domestic violence, while blue areas indicate a negative effect, with darker shades representing stronger impacts. **(a)** age group 20–40 years in 2020; **(b)** age group 40–65 years in 2020; **(c)** age group 20–40 years in 2021; **(d)** age group 40–65 years in 2021; **(e)** age group 20–40 years in 2022; and **(f)** age group 40–65 years in 2022.

**Figure 8:**
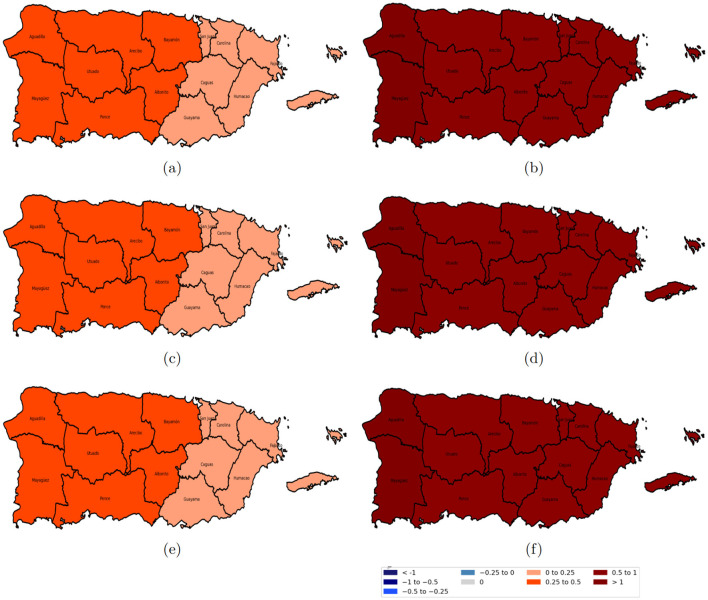
Spatial variation of the education level and female coefficients from 2020 to 2022. Red areas indicate a positive effect of education level on domestic violence, while blue areas indicate a negative effect, with darker shades representing stronger impacts. **(a)** population with a bachelor's degree or higher in 2020; **(b)** female population in 2020; **(c)** population with a bachelor's degree or higher in 2021; **(d)** female population in 2021; **(e)** population with a bachelor's degree or higher in 2022; and **(f)** female population in 2022.

**Figure 9:**
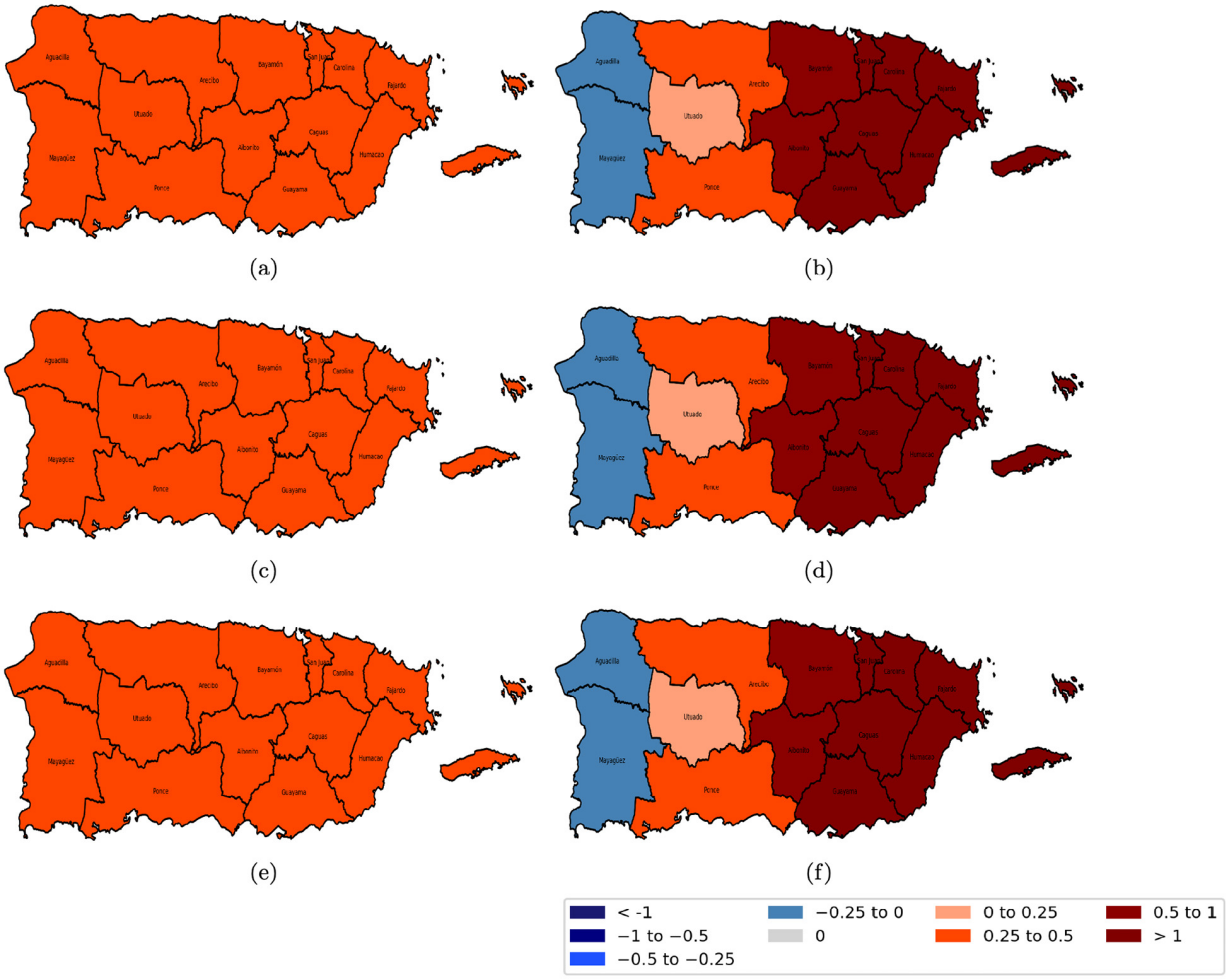
Spatial variation of the marital status (divorced and separated) coefficients from 2020 to 2022. Red areas indicate a positive effect of marital status on domestic violence, while blue areas indicate a negative effect, with darker shades representing stronger impacts. **(a)** divorced population in 2020; **(b)** separated population in 2020; **(c)** divorced population in 2021; **(d)** separated population in 2021; **(e)** divorced population in 2022; and **(f)** separated population in 2022.

**Figure 10:**
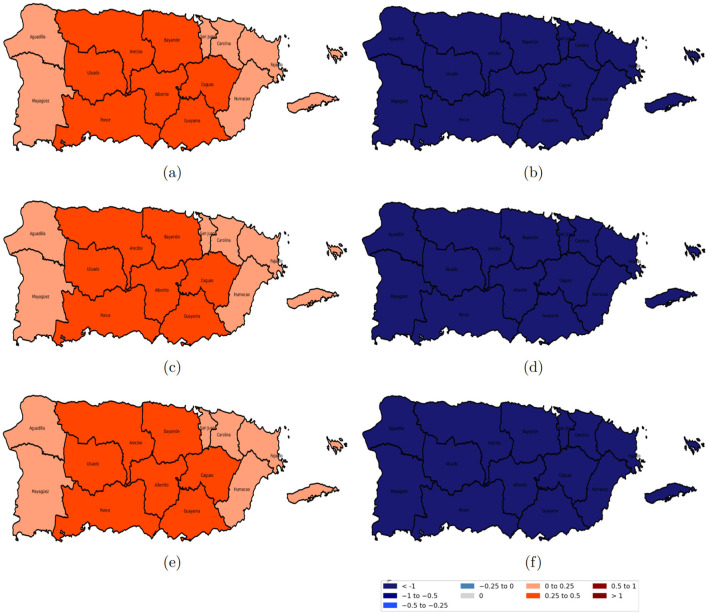
Spatial variation of the estimated coefficients for the social isolation index and moderate income stress from 2020 to 2022. Red areas indicate a positive effect on domestic violence, while blue areas indicate a negative effect, with darker shades representing stronger impacts. **(a)** social isolation index in 2020; **(b)** moderate income stress in 2020; **(c)** social isolation index in 2021; **(d)** moderate income stress in 2021; **(e)** social isolation index in 2022; and **(f)** moderate income stress in 2022.

Figures 7–10 illustrate the spatiotemporal variations in the relationships between reported domestic violence cases and various socioeconomic predictors from 2020 to 2022 from the GTWR model. The coefficient maps are displayed using a standardized classification scheme to facilitate interpretation of effect magnitude and direction across space and time. The positive coefficients are categorized into weak (0 to 0.25), moderate (0.25 to 0.5), and strong (0.5 to 1 and >1.0), while negative coefficients are similarly classified into weak (−0.25 to 0), moderate (−0.5 to −0.25), and strong (−1 to −0.5 and < −1); coefficients equal to zero indicate no local association.

A summary of local GTWR coefficient distributions (mean, standard deviation, range, and percentage of statistically significant regions) is provided in Appendix Table 1. Overall, coefficients exhibited substantial spatial heterogeneity for selected predictors, particularly age 20–40 years, moderate income stress, and educational attainment, while others showed weaker or more spatially uniform associations. Notably, the magnitude and dispersion of local coefficients remained highly consistent across 2020-2022, indicating temporal stability in the spatial structure of associations despite year-to-year variation in reported domestic violence levels.

The relationship between the population aged 40 to 65 in Figures 7b, d, f and domestic violence cases was predominantly negative across most police regions in Puerto Rico, except for Fajardo and Humacao, where a positive association was observed. In contrast, the population aged 20–40 showed a strong positive association across all police regions, as shown in Figures 7a, c, e.

Figure 8 shows the coefficients of educational attainment and the female population; the population with a bachelor's degree or higher in Figures 8a, c, e had a slightly positive association with domestic violence in the eastern region, which became stronger in the western region. Conversely, the female population in Figures 8b, d, f consistently showed a strong positive relation with domestic violence across all regions and years.

The divorced population, as illustrated in Figures 9a, c, e, was positively associated with reported cases of domestic violence. However, for the separated population, the relationship was spatially heterogeneous: a negative relationship was observed in the western regions (specifically Aguadilla and Mayagüez), a slightly positive correlation in central Puerto Rico, and a strong positive correspondence in the eastern regions as shown in Figures 9b, d, f.

Figures 10a, c, e, the social isolation index was positively related to domestic violence, with the strongest relationships observed in the central regions of Puerto Rico and weaker positive correspondence along the eastern and western areas. In contrast, moderate income stress in Figures 10b, d, f was negatively correlated with domestic violence, indicating that higher levels of moderate income stress were linked with lower domestic violence rates.

The GTWR resulting optimal bandwidths were *b* = 74.8 for the spatial kernel and τ = 3.2 for the temporal kernel, indicating the effective spatial and temporal ranges over which observations exert influence in the calibration. Given that the temporal domain spans annual observations from 2020 to 2022, the temporal bandwidth (τ = 3.2) implied that information from neighboring years contributed substantially to local parameter estimation, reflecting smooth temporal evolution in domestic violence dynamics. The spatial bandwidth (*b* = 74.8 km) indicated that observations within approximately 75 km exerted the greatest influence on local coefficient estimates, capturing regional-scale spatial dependence across adjacent police regions.

Model performance metrics for the ordinary least squares (OLS) and the geographically and temporally weighted regression (GTWR) models are summarized in Table 7. The GTWR model demonstrated a notable improvement over the OLS model. Specifically, the adjusted R^2^ increased from 0.4948 (OLS) to 0.5626 (GTWR), while the root mean square error (RMSE) decreased from 0.6410 to 0.5634. Although the corrected Akaike information criterion (AICc) remained the same at 1040 for both models, the improvements in adjusted R^2^ and RMSE suggested better model fit under the GTWR.

**Table 7:** Performance evaluation of OLS and GTWR models.

Model	*R* ^2^	Adjusted *R*^2^	AICc	RMSE
OLS	0.6012	0.4948	100.1984	0.6410
GTWR	0.6919	0.5626	100.6611	0.5634

AICc represents the Akaike Information Criterion corrected, and RMSE represents the root mean square error.

This diagnostic supported the suitability of the OLS model as a baseline comparison.

**Figure 11:**
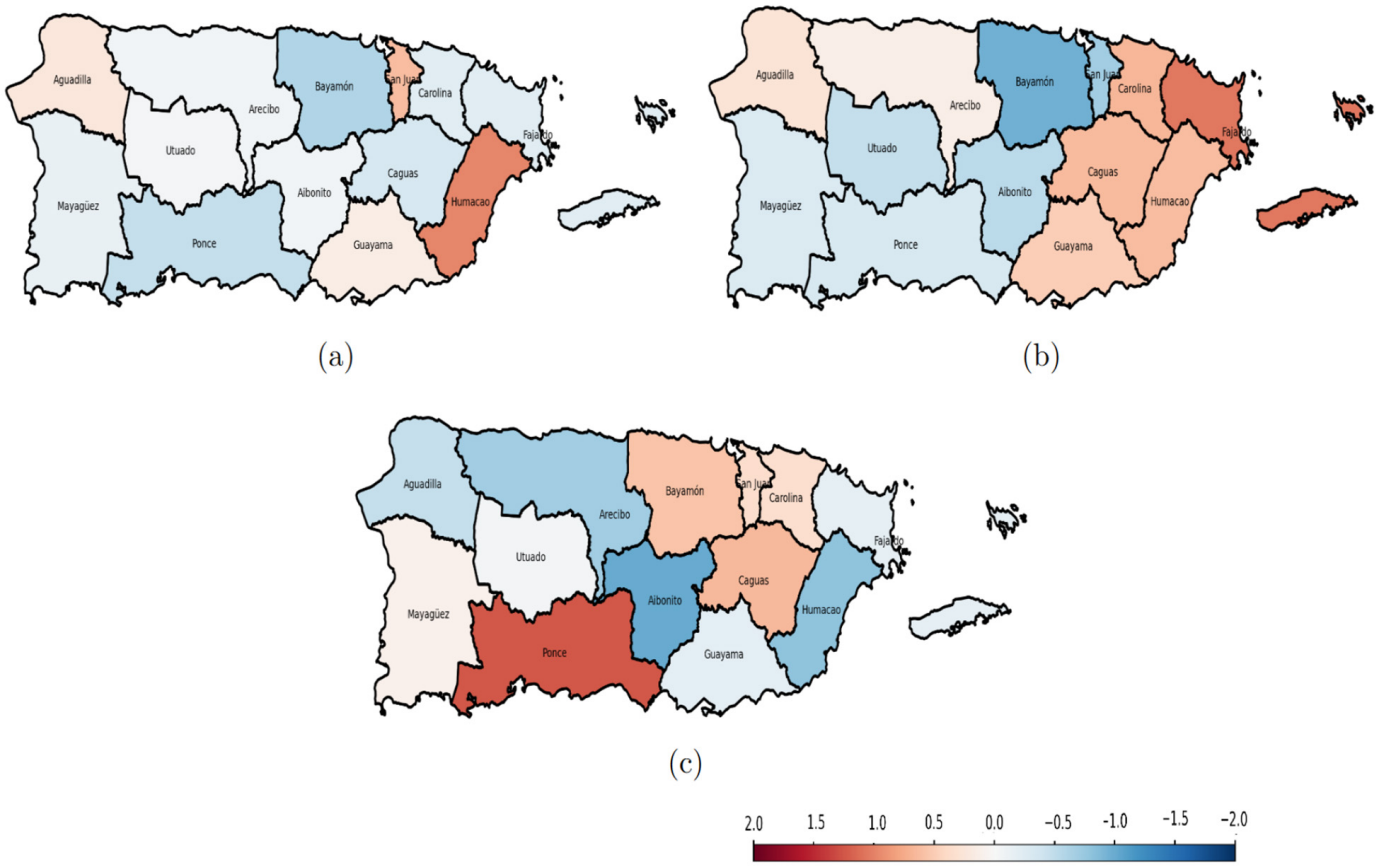
Spatial variation of the residuals of the GTWR model from 2020 to 2022. Red areas indicate positive and blue areas indicate negative. **(a)** 2020 GTWR residuals; **(b)** 2021 GTWR residuals; **(c)** 2022 GTWR residuals.

The residual maps for 2020 to 2022 in Figure 11 show that most residual values are close to zero, indicating that the GTWR model provided a good fit across Puerto Rico. These near-zero residuals suggest that predicted domestic violence rates closely aligned with the observed values in most police regions, with only a few localized areas exhibiting notable over- or under-predictions. To further evaluate whether the model adequately accounted for spatial dependence, spatial autocorrelation in the model residuals was assessed using global Moran's I statistics. Overall residual spatial autocorrelation across all years was minimal and statistically non-significant (Moran's I = 0.0245, *p* = 0.133), indicating that GTWR substantially reduced the spatial clustering present in the observed domestic violence data.

In summary, minimal temporal variation was observed, likely due to the limited number of years covered in the study.

## 4 Discussion and conclusion

### 4.1 Discussion

Domestic violence is a major public health concern in Puerto Rico, with 17,009 police-reported cases recorded between 2020 and 2022. In 2023, the Puerto Rican Government Office of the Women's Advocate reported a further 42.7% increase in reported incidents (45), underscoring the urgency of understanding how domestic violence patterns evolve across space and time.

We examine police-reported domestic violence during the COVID-19 pandemic (2020–2022), a period marked by strict mobility restrictions and documented changes in population movement. Using geographically and temporally weighted regression (GTWR), we assess how age structure, education, marital status, moderate income stress, and social isolation are associated with domestic violence patterns across Puerto Rico's thirteen police regions while explicitly modeling spatial and temporal heterogeneity.

This study makes three contributions. First, we show that associations between socioeconomic conditions and police-reported domestic violence are spatially and temporally non-stationary within Puerto Rico, indicating that global models mask meaningful regional variation. We then demonstrate GTWR model performance relative to ordinary least squares. Finally, translate the localized coefficients into policy-relevant quantitative offsets and assess robustness.

The data in Figure 2 showed a higher number of reported domestic violence cases in 2021 compared to other years. This is consistent with global trends observed during the COVID-19 pandemic, where domestic violence cases increased significantly in response to mobility restrictions and social stressors (46). Previous research has documented notable increases in domestic violence during this time (47–50), which could be attributed to pandemic-related stressors such as mobility restrictions and stay-at-home orders, increased economic and social pressures, higher unemployment, and reduced access to social support networks and community services. These conditions likely intensified household tensions and limited opportunities for victims to seek assistance, contributing to the rise in reported cases.

The substantial decline in 2020 contrasts sharply with global expectations and underscores the need for investigation into the underlying factors. Victims may have faced greater barriers to accessing reporting mechanisms during lockdowns (51). Local policies and law enforcement practices may have influenced both the prevalence and recording of domestic violence cases. Additionally, social support interventions such as community outreach programs or increased availability of remote counseling services could have been connected with changes in reported risk patterns (52). Cultural or societal differences in household dynamics and resilience may also have been linked with observed patterns.

These evolving spatial patterns may have been associated with shifts in local policy responses, community-level interventions, or law enforcement practices during and after the COVID-19 pandemic. The High–Low pattern observed in Ponce may indicate localized risk factors not present in neighboring regions, or alternatively, lower reporting levels in surrounding areas, possibly due to differences in access to support services or reporting mechanisms (53). Meanwhile, the sustained High–High clustering in San Juan and Bayamón highlights regions where structural and socio-economic vulnerabilities may be linked with elevated domestic violence rates.

To better capture the spatiotemporal dynamics of domestic violence, this study applied the GTWR regression model. Compared with the traditional OLS regression, the GTWR model substantially improved both explanatory power and model fit. These findings underscored the limitations of classical statistical methods when spatial and temporal dependencies are not adequately addressed.

The GTWR coefficient estimates revealed several important patterns in the relationship between socioeconomic factors and reported domestic violence cases. Notably, education showed a predominantly positive association across all years, with populations with a bachelor's degree or higher. In rural Turkey increase in education among women led to more reporting of domestic violence incidents (54), and in Bangladesh, women with higher education in comparison to their male spouses were more likely to experience DV (55). correspondence. This is consistent with previous studies suggesting that an increase in education is positively associated with domestic violence, which may be in part due to resource based willingness to report.

The spatial variation in the coefficients further highlighted that the relation of the population aged 20-40 years, those aged 40-65 years, and the maritally separated population is not uniform across regions. These differences may be associated with variations in local social structures, cultural norms, and the availability of social services. Consistent with this evidence, our findings show that the population aged 20–40 years is positively associated with domestic violence across Puerto Rico.

Similarly, the spatial heterogeneity observed in the relationship between the separated marital status population and domestic violence emphasizes the need for region-specific interventions. The western part of the island shows a negative relationship with domestic violence among separated couples, in contrast to a progressively increasing positive correspondence moving eastward.

The social isolation index also demonstrated significant spatiotemporal patterns. The positive association between social isolation and domestic violence across the island suggests that individuals or households experiencing greater isolation are at higher risk. Beyond its association with reported cases, the social isolation index represents a methodological contribution by operationalizing population-level mobility contraction using multi-source data streams. Incorporating this contextual proxy into GTWR enabled explicit modeling of crisis-period confinement dynamics, which are often theorized but rarely quantified in spatial domestic violence research. Interestingly, regions with higher proportions of middle-income populations showed a strong negative relationship with domestic violence, while in the female population, a strong positive association was observed.

To translate model associations into an interpretable scale, we computed standardized offset values from the GTWR linear predictor. For a standardized predictor *X*_*k*_ with local coefficient β_*k*_, the modeled change in police-reported domestic violence (Δ*Y*) associated with a change Δ*X*_*k*_ is approximated as Equation 11


$$\begin{array}{c} \Delta Y\approx \beta_{k}\Delta X_{k}. \end{array}$$ (11)

Rearranging yields the standardized change required to counterbalance a modeled shift in police-reported cases Equation 12:


$$\begin{array}{c} \Delta X_{k}^{\text{offset}}=-\frac{\Delta Y}{\beta_{k}}. \end{array}$$ (12)

Because all predictors were standardized, $\Delta X_{k}^{\text{offset}}$ is expressed in standard deviation (SD) units. These values represent linear approximations derived from the fitted model and do not imply causal effects.

Table 8 shows that predictors with larger absolute coefficients require smaller standardized shifts to offset modeled variation in reported cases. Moderate income stress ($\bar{\beta }=-8.02$) exhibits the greatest modeled leverage: a small standardized change (median ≈0.054 SD) corresponds to the predicted change in police-reported domestic violence. In contrast, education and marital composition variables require substantially larger shifts (approximately 0.28-1.0 SD), indicating comparatively weaker modeled influence. Age 40–65 years shows minimal leverage, requiring large standardized adjustments to produce comparable modeled change.

**Table 8:** Model-implied standardized offset values required to counterbalance mean predicted changes in police-reported domestic violence (DV).

Variable	Mean β	Mean pred. change	Median offset (SD)	P25	P75
Moderate income stress	−8.003	−4.001	0.053	0.051	0.055
Age 20–40 years	2.904	1.452	−0.150	−0.168	−0.131
Separated population	1.378	0.689	−0.214	−0.342	−0.146
Divorced population	0.532	0.267	−0.847	−0.867	−0.722
Bachelor's or higher	0.458	0.229	−1.017	−1.236	−0.730
Age 40–65 years	−0.155	−0.078	1.675	1.124	4.706
Female population	1.545	0.772	−0.281	−0.290	−0.255

Offsets are expressed in standard deviation (SD) units of each predictor. Results are based on 39 region-year observations.

These offsets provide a scale for comparing the magnitude of spatially varying associations, clarifying which socioeconomic dimensions exhibit stronger modeled relationships with police-reported domestic violence while remaining consistent with the observational nature of the data.

### 4.2 Conclusion

This study confirmed the spatial and temporal distribution of police-reported domestic violence cases at the police-region scale (13 regions) in Puerto Rico from 2020 to 2022. Geographically and temporally weighted regression (GTWR) was employed to examine the relationship between selected socioeconomic indicators and police-reported domestic violence. While the multifaceted nature of domestic violence cannot be fully captured by socioeconomic variables alone, the findings provide quantitative evidence of spatial heterogeneity and temporal stability in these associations at a finer regional scale. Several predictors, including the proportion of individuals aged 40–65 years, educational attainment and the separated population, exhibited consistent spatial variation across regions, with distinct patterns between western and eastern portions of the island. These spatial disparities underscore the importance of localized characterization of communities when considering prevention strategies and resource allocation. Rather than implying causal effects, the results identify region-specific associations that may inform place-based and time-sensitive policy considerations.

Notwithstanding these contributions, several limitations must be acknowledged. The analysis is based on 39 region-year observations (13 police regions observed annually from 2020 to 2022), which constrains statistical power and the complexity of additional modeling strategies. The outcome variable reflects police-reported domestic violence cases and therefore likely underestimates true incidence. Reporting levels may vary across regions and over time due to differences in institutional capacity, mobility restrictions, service access, and other crisis-related disruptions during the COVID-19 period. Although scenario-based sensitivity analyses assuming 10%, 25%, and 40% underreporting yielded stable coefficient directions across predictors, observed spatial-temporal patterns may still partially reflect reporting dynamics rather than underlying changes in violence.

Future research could integrate alternative data sources, including victimization surveys, emergency call records, hotline utilization data, and protection order filings, to better distinguish reporting processes from underlying violence. Expanding the temporal window to include additional pre-pandemic years would also strengthen baseline comparisons and allow more comprehensive assessment of crisis-related shifts in police-reported domestic violence. Increasing the spatial window using the municipality-level domestic violence records would allow more granular spatial modeling and potentially stronger detection of localized heterogeneity.

## Data Availability

Publicly available datasets were analyzed in this study. This data can be found here: Domestic violence cases from the Puerto Rico Police Bureau https://app.powerbigov.us/view?r=eyJrIjoiNWI4ZjI0ZTItY2Y3NC00MTJjLTg3ZjctNjA3YjJlNmE1MTc1IiwidCI6ImUwYzIyNzAyLTA5MmYtNGRhYi1hNTkyLWZhYjUyZGRlNGMxZiJ9; Census Bureau https://www.census.gov/geographies/mapping-files/time-series/geo/tiger-line-file.html.

## Appendix

**Table A1:** Summary statistics of local GTWR coefficients by year (baseline model).

Year	Variable	Mean	SD	Min	Max	% Sig.	Mean|β|
2020	20–40 Years	1.7424	0.2757	1.4311	2.2928	100	1.7424
	40–65 Years	−0.0930	0.0851	−0.2187	0.0513	0	0.1026
	Divorced Population	0.3193	0.0549	0.2537	0.4212	100	0.3193
	Female Population	0.9271	0.0986	0.8476	1.1195	100	0.9271
	Moderate Income Stress	−4.8023	0.3699	−5.5450	−4.5062	100	4.8023
	Separated Population	0.8270	0.6510	−0.0875	1.9404	0	0.8529
	Isolation Index	0.2467	0.0114	0.2198	0.2594	0	0.2467
	Bachelor's or Higher	0.2750	0.1071	0.1228	0.4531	46.1538	0.2750
2021	20–40 Years	1.7421	0.2756	1.4309	2.2924	100	1.7421
	40–65 Years	−0.0930	0.0851	−0.2188	0.0513	0	0.1027
	Divorced Population	0.3193	0.0549	0.2537	0.4212	100	0.3193
	Female Population	0.9269	0.0986	0.8474	1.1192	100	0.9269
	Moderate Income Stress	−4.8020	0.3699	−5.5447	−4.5058	100	4.8020
	Separated Population	0.8265	0.6509	−0.0878	1.9399	0	0.8525
	Isolation Index	0.2468	0.0114	0.2199	0.2595	0	0.2468
	Bachelor's or Higher	0.2749	0.1071	0.1227	0.4530	46.1538	0.2749
2022	20–40 Years	1.7418	0.2756	1.4307	2.2921	100	1.7418
	40–65 Years	−0.0931	0.0851	−0.2188	0.0512	0	0.1027
	Divorced Population	0.3193	0.0549	0.2537	0.4211	100	0.3193
	Female Population	0.9266	0.0986	0.8471	1.1190	100	0.9266
	Moderate Income Stress	−4.8017	0.3699	−5.5444	−4.5055	100	4.8017
	Separated Population	0.8260	0.6509	−0.0882	1.9394	0	0.8521
	Isolation Index	0.2469	0.0114	0.2200	0.2596	0	0.2469
	Bachelor's or Higher	0.2749	0.1071	0.1227	0.4529	46.1538	0.2749

Values represent regional means (n = 13 police regions per year), dispersion, and percentage of statistically significant local coefficients.

Mean|β| denotes the mean absolute value of local coefficients across regions.

% Sig. indicates the percentage of police regions with statistically significant local coefficients at the chosen significance level.

## References

[1] Delgadillo-Aleman S Ku-Carrillo R Perez-Amezcua B Chen-Charpentier B A. mathematical model for intimate partner violence. Mathem Comput Appl. (2019) 24:29. doi: 10.3390/mca24010029

[2] Flowers RB. Domestic Crimes, Family Violence and Child Abuse: A Study of Contemporary American Society. Jefferson, NC: McFarland. (2000).

[3] Cullen C. Method matters: the underreporting of intimate partner violence. World Bank Econ Rev. (2022) 37:49–73. doi: 10.1093/wber/lhac022

[4] Ohajunwa C Caiseda C. Computational modeling, analysis and simulation for lockdown dynamics of COVID-19 and domestic violence. Elect Res Archive. (2022) 30:2446–2464. doi: 10.3934/era.2022125

[5] World Health Organization. Who director-general's opening remarks at the media briefing on covid-19. (2020). Available online at: https://www.who.int/ (Acessed March 14, 2025).

[6] Government of Puerto Rico. Executive Order oe-2020-023: To Implement a Curfew and Close Non Essential Businesses Due to Covid-19. (2020). Available online at: https://www.estado.pr.gov/en/executive-orders/ (Acessed February 14, 2025).

[7] Puerto Rico Department of Health. Covid-19 Public Health Response and Situation Reports in Puerto Rico. (2020). Available online at: https://www.salud.gov.pr/ (Acessed March 14, 2025).

[8] Centers for Disease Control and Prevention. Covid-19 Response in Puerto Rico. (2020). Available online at: https://www.cdc.gov/coronavirus/ (Acessed March 10, 2025).

[9] Zhang H. The influence of the ongoing COVID-19 pandemic on family violence in China. J Fam Violence. (2022) 37:733–43. doi: 10.1007/s10896-020-00196-8 32921903 PMC7473410

[10] Bettinger-Lopez C Bro A A. double pandemic: Domestic violence in the age of COVID-19. New York: Council on Foreign Relations. (2020) 13:1–7.

[11] Deese K. Divorces Skyrocket in China Amid Lockdown. Washington, DC: The Hill. (2020) 1:2020.

[12] Hossain MM Asadullah M Rahaman A Miah MS Hasan MZ Paul T . Prediction on domestic violence in bangladesh during the covid-19 outbreak using machine learning methods. Applied Syst Innovat. (2021) 4:77. doi: 10.3390/asi4040077

[13] Evans DP Hawk SR Ripkey CE. Domestic violence in Atlanta, Georgia before and during COVID-19. Viol Gender. (2021) 8:140–7. doi: 10.1089/vio.2020.0061 34466626 PMC8403185

[14] Hsieh T Wang YH Hsieh YS Ke JT Liu CK Chen SC. Measuring the unmeasurable—a study of domestic violence risk prediction and management. J Technol Hum Serv. (2018) 36:56–68. doi: 10.1080/15228835.2017.1417953

[15] Maposa I Twabi HS Matsena-Zingoni Z Batidzirai JM Singini G Mohammed M . Bayesian spatial modelling of intimate partner violence and associated factors among adult women and men: evidence from 2019/2020 Rwanda Demographic and Health Survey. BMC Public Health. (2023) 23:2061. doi: 10.1186/s12889-023-16988-8 37864202 PMC10589974

[16] Elaine Enarson. Violence against women in disasters. Viol Against Women. (1999) 5:742–768. doi: 10.1177/10778019922181464

[17] Fisher S. Violence against women and natural disasters: findings from post-disaster contexts. Viol Against Women. (2010) 16:902–18. doi: 10.1177/1077801210377649 20679186

[18] Hrabok M Delorme A Agyapong V. Threats to mental health and well-being associated with climate change. J Anxiety Disord. (2020) 76:102295. doi: 10.1016/j.janxdis.2020.102295 32896782

[19] Gearhart S Perez-Patron M Hammond TA Goldberg DW Klein A Horney JA. The impact of natural disasters on domestic violence: An analysis of reports of simple assault in Florida (1999–2007). Viol Gender. (2018) 5:87–92. doi: 10.1089/vio.2017.0077

[20] Harville EW Taylor CA Tesfai H Xiong X Buekens P. Experience of hurricane Katrina and reported intimate partner violence. J Interpers Viol. (2011) 26:833–45. doi: 10.1177/0886260510365861 20495099 PMC3472442

[21] Matheson K Asokumar A Anisman H. Resilience: safety in the aftermath of traumatic stressor experiences. Front Behav Neurosci. (2020) 14:596919. doi: 10.3389/fnbeh.2020.596919 33408619 PMC7779406

[22] Rivera FI Rolke W. Uneven recovery after hurricane maria: Evidence from Puerto Rico. Nat Hazards Rev. (2018) 19:04018013. doi: 10.1002/sim.8314

[23] García C Rivera FI Garcia MA Burgos G Aranda MP. Contextualizing the COVID-19 era in Puerto Rico: Compounding disasters and parallel pandemics. J Gerontol: Series B. (2021) 76:e263–e267. doi: 10.1093/geronb/gbaa186 33112945 PMC7665778

[24] Puerto Rico. (2024). Available online at: https://www.usgs.gov/programs/national-geospatial-program/news/usgs-releases-new-topographic-maps-puerto-rico-and-us (Acessed March 14, 2025).

[25] Police Regions. (2024). Available online at: https://dbpedia.org/page/Puerto_Rico_Police (Accessed March 14, 2025).

[26] Peterman A Potts A O'Donnell M Thompson K Shah N Oertelt-Prigione S . Pandemics and violence against women and children. In: Center for Global Development Working Paper. Washington, DC: Center for Global Development (2020). p. 528.

[27] Socioeconomic and Demographic Datasets. (2023). Available online at: https://data.census.gov/table/ACSDP1Y2017.DP05?q=United+States&table=DP05&g=010XX00US&lastDisplayedRow=29&vintage=2017&layer=state&cid=DP05_0001E&tid=ACSDP1Y2017.DP05 (Accessed March 14, 2025).

[28] Shapefile Data. (2023). Available online at: https://www.census.gov/geographies/mapping-files/time-series/geo/tiger-line-file.html (Accessed March 14, 2025).

[29] Google. COVID-19 Community Mobility Reports. (2025). Available online at: https://www.google.com/covid19/mobility/ (Accessed August 27, 2025).

[30] Gobierno de Puerto Rico. Generación, Consumo, Costo, Ingresos y Clientes del Sistema Eléctrico de Puerto Rico. (2025). Available online at: https://indicadores.pr/dataset/generacion-consumo-costo-ingresos-y-clientes-del-sistema-electrico-de-puerto-rico (Accessed August 27, 2025).

[31] Meta Platforms Inc (Facebook Data for Good). Movement-Range Maps. (2025). Available online at: https://dataforgood.facebook.com/dfg/tools/movement-range-maps/ (Accessed August 27, 2025).

[32] GTWR. (2022). Available online at: https://github.com/sunkun1997/mgtwr (Accessed March 14, 2025).

[33] Hance EJD Rodríguez MT Caraballo FMA. Impact of mitigation strategies: The case of the covid-19 timeline in Puerto Rico. Revista [IN]Genios. (2021) 7(2):1–16

[34] Chubb H Simpson JM. The use of Z-scores in paediatric cardiology. Ann Pediatr Cardiol. (2012) 5:179–84. doi: 10.4103/0974-2069.99622 23129909 PMC3487208

[35] Moran PA. Notes on continuous stochastic phenomena. Biometrika. (1950) 37:17–23. doi: 10.1093/biomet/37.1-2.17 15420245

[36] Li X Anselin L. RGEODA: R Library for Spatial Data Analysis, 2025. R Package Version 0.1.0.

[37] Montgomery DC Peck EA Vining GG. Introduction to linear regression analysis. Hoboken, NJ: John Wiley & Sons. (2021).

[38] Fotheringham AS Crespo R Yao J. Geographical and temporal weighted regression (GTWR). Geogr Anal. (2015) 47:431–52. doi: 10.1111/gean.12071

[39] Brunsdon C Fotheringham S Charlton M. Geographically weighted regression. J Royal Statist Soc. (1998) 47:431–43. doi: 10.1111/1467-9884.00145

[40] Roure JG. Gender justice in Puerto Rico: Domestic violence, legal reform, and the use of international human rights principles. Hum Rights Q. (2011) 33:790–825. doi: 10.1353/hrq.2011.0042

[41] Anselin L. Local indicators of spatial association—LISA. Geogr Anal. (1995) 27:93–115. doi: 10.1111/j.1538-4632.1995.tb00338.x

[42] Dong F Zhang S Long R Zhang X Sun Z. Determinants of haze pollution: An analysis from the perspective of spatiotemporal heterogeneity. J Clean Prod. (2019) 222:768–83. doi: 10.1016/j.jclepro.2019.03.105

[43] Muthukrishnan R Rohini R. Lasso: a feature selection technique in predictive modeling for machine learning. In: 2016 IEEE International Conference on Advances in Computer Applications (ICACA). Coimbatore: IEEE (2016). p. 18–20. doi: 10.1109/ICACA.2016.7887916

[44] Breusch TS Pagan AR. A simple test for heteroscedasticity and random coefficient variation. Econometrica. (1979) 47:1287–1294. doi: 10.2307/1911963

[45] de la Procuradora de la Mujer O. Informe anual: 1 de enero de 2023 al 30 de junio de 2024 *Gobierno de Puerto Rico*. (2024). Available online at: https://docs.pr.gov/files/Mujer/Informes/Informe%20Anual%20OPM%202023-2024.pdf (Acessed August 27, 2025).

[46] Piquero AR Jennings WG Jemison E Kaukinen C Knaul FM. Domestic violence during the COVID-19 pandemic-Evidence from a systematic review and meta-analysis. J Crim Justice. (2021) 74:101806. doi: 10.1016/j.jcrimjus.2021.101806 36281275 PMC9582712

[47] Boserup B McKenney M Elkbuli A. Alarming trends in US domestic violence during the COVID-19 pandemic. Am J Emerg Med. (2020) 38:2753. doi: 10.1016/j.ajem.2020.04.077 32402499 PMC7195322

[48] Neil J. Domestic violence and COVID 19. Australian Journal for General Practitioners. (2020). 49. doi: 10.31128/AJGP-COVID-25 32539247

[49] Peitzmeier SM Fedina L Ashwell L Herrenkohl TI Tolman R. Increases in intimate partner violence during COVID-19: Prevalence and correlates. J Interpers Violence. (2022) 37:NP20482–512. doi: 10.1177/08862605211052586 34866451 PMC9014340

[50] Usta J Murr H El-Jarrah R. COVID-19 lockdown and the increased violence against women: understanding domestic violence during a pandemic. Viol Gender. (2021) 8:133–9. doi: 10.1089/vio.2020.0069

[51] Speed A Thomson C Richardson K. Stay home, stay safe, save lives? An analysis of the impact of COVID-19 on the ability of victims of gender-based violence to access justice. J Criminal Law. (2020) 84:539–72. doi: 10.1177/0022018320948280

[52] Wasserman D Iosue M Wuestefeld A Carli V. Adaptation of evidence-based suicide prevention strategies during and after the COVID-19 pandemic. World Psychiatry. (2020) 19:294–306. doi: 10.1002/wps.20801 32931107 PMC7491639

[53] de Souza Santos D Bittencourt EA de Moraes Malinverni AC Kisberi JB de França Vilaça S Iwamura ESM. Domestic violence against women during the Covid-19 pandemic: a scoping review. Forensic Sci Int: Rep. (2022) 5:100276. doi: 10.1016/j.fsir.2022.100276 38013975 PMC9125991

[54] Erten B Keskin P. For better or for worse?: Education and the prevalence of domestic violence in turkey. Am Econ J. (2018) 10:64–105. doi: 10.1257/app.20160278

[55] Rapp D Zoch B Khan MH Pollmann T Krämer A. Association between gap in spousal education and domestic violence in India and Bangladesh. BMC Public Health. (2012)12:467. doi: 10.1186/1471-2458-12-467 22720800 PMC3490925

[56] Median Household Income. (2023). Available online at: https://data.census.gov/table?t=Income+(Households,+Families,+Individuals)&g=040XX00US72,720500000 (accessed March 14, 2025).

[57] Age Distribution. (2023). Available online at: https://data.census.gov/table?t=Age+and+Sex&g=040XX00US72,720500000 (accessed March 14, 2025).

[58] Female Population. (2023). Available online at: https://data.census.gov/table?t=Age+and+Sex&g=040XX00US72,720500000 (accessed March 14, 2025).

[59] Education Level. (2023). Available online at: https://data.census.gov/table?t=Educational+Attainment&g=040XX00US72,720500000 (accessed March 14, 2025).

[60] Reported Domestic Violence Cases. (2022). Available online at: https://app.powerbigov.us/ (accessed August 27, 2025).

[61] Census Data. (2023). Available online at: https://data.census.gov/table (accessed March 14, 2025).

[62] Bureau PRP. Domestic Violence Statistics. (2025). Available online at: https://app.powerbigov.us/view?r=eyJrIjoiNWI4ZjI0ZTItY2Y3NC00MTJjLTg3ZjctNjA3YjJlNmE1MTc1IiwidCI6ImUwYzIyNzAyLTA5MmYtNGRhYi1hNTkyLWZhYjUyZGRlNGMxZiJ9 (accessed September 25, 2025).

